# Effect of microsurgical varicocelectomy on semen parameters, serum, and intratesticular testosterone levels

**DOI:** 10.1002/bco2.15

**Published:** 2020-05-04

**Authors:** Thiago Fernandes Negris Lima, Fabio Stefano Frech, Premal Patel, Ruben Blachman-Braun, Ranjith Ramasamy

**Affiliations:** 1 Department of Urology, Miller School of Medicine, University of Miami, Miami, FL, USA; 2 Department of Urology, University of Manitoba, Winnipeg, MB, Canada

**Keywords:** 17-hydroxyprogesterone, intratesticular testosterone, semen analysis, testosterone, varicocele

## Abstract

**Objective:**

The goal of this work was to evaluate if men who underwent microsurgical varicocelectomy would have improvement in serum testosterone (T) as well as serum 17-hydroxyprogesterone (17-OHP—An intratesticular T biomarker) in addition to semen parameters after operation.

**Materials and Methods:**

We conducted a prospective analysis of 30 men who underwent microsurgical varicocelectomy from December 2018 to September 2019. We assessed varicocele grade and laterality, serum T, serum 17-OHP, serum follicle- stimulating hormone (FSH), serum LH, and semen parameters in baseline and follow-up. According to the data distribution, we reported the median and interquartile ranges and utilized the Mann-Whitney U, Student’s *t* test and Wilcoxon rank test. Correlation analysis was performed with the Spearman test.

**Results:**

In the baseline, 9 (30%) men had 17-OHP < 55 ng/dL and 21 (70%) men presented with 17-OHP > 55 ng/dL. Also, 19 men had TMSC < 9 million, including 6 men with azoospermia, 1 man with cryptozoospermia, and 11 men with TMSC > 9 million. We found an improvement in most SA parameters of most men, which include concentration (63.3%, 19/30), motility (46.6%, 14/30), and TMSC (60%, 18/30). About seven (36.8%) men had TMSC upgraded to > 9. There was a significant change in volume (2.1 [1.5–2.8] to 2.4 [1.7–3.6] cc, *P* = .018), concentration (6.8 [0.8–22.5] to 12.5 [1–31] million/cc, *P* = .047) and TMSC (4.4 [0.3–15.1] to 10.5 [0–41.8] million, *P* = .012) after surgery. We neither found a change in serum T nor a change in intratesticular T (serum 17-OHP) after varicocelectomy (*P* > .05). FSH, LH and T were similar both before and after varicocelectomy (*P* > .05).

**Conclusion:**

Despite improvement in semen parameters following varicocelectomy, we did not see changes in either serum or intratesticular T. This suggests that improvement of semen parameters following varicocele repair could be from factors other than changes in androgen levels within the testis.

## 1| INTRODUCTION

Varicocele, defined as abnormally enlarged and tortuous veins of the pampiniform plexus, is the most common identified cause of male factor infertility. It is found in 15% of the general population, but is present in up to 35% of men presenting with primary infertility and up to 80% of men presenting with secondary infertility.^1^ Varicocele can negatively impact appropriate sperm production leading to decreased fertility and complete azoospermia in 4%−14% of patients.^2^ The proposed detrimental effects of varicocele on testicular function include oxidative stress, high scrotal temperature with loss of the scrotal counter-cooling mechanism, reflux of adrenal metabolites, and changes in androgen production.^3,4^

Leydig cells drive spermatogenesis via the secretion of testosterone (T), which acts on Sertoli and/or peritubular cells to create an environment which enables normal progression of germ cells through the spermatogenic cycle.^5^ Also, testicular biopsy in patients with idiopathic varicocele showed decreased tubular diameter, increased Leydig cell atrophy with vacuolization and decreased Leydig cell number in testicular tissue when staining for T.^6^ These studies suggest that varicoceles affect Leydig cell histology, and thus, likely affecting sperm production and T levels. Although little data has been published, some authors showed an association between varicocele and low T, and subsequent improvement after surgical repair.^7,8,9,10^ However, a large population study among adolescents showed no association between varicocele and serum T levels.^11^

We based the correlation of 17-OHP with ITT in Amory et al^12^ study, that compared ITT levels through testicular aspiration with serum 17-OHP levels in men previously treated with exogenous T who were distributed in different hCG dosages groups (*r* = .78, *P* < .0001). We published a recent study in *J Urol*^13^ evaluating the positive association between 17-OHP and ITT in a study comparing men receiving exogenous T to men receiving clomiphene citrate or human chorionic gonadotropin, where men receiving exogenous T had undetectable 17-OHP levels in follow-up, while men receiving medications to increase ITT had a significant increase in 17-OHP levels. Since one of the mechanisms by which varicocelectomy improves semen parameters is with improvement in ITT production, we hypothesized that semen analysis parameters improvement after varicocele repair could be associated to augmentation of ITT levels. The objective of this study was to evaluate whether changes in semen parameters following varicocele repair will be associated with changes in either ITT or serum T.

## 2 | MATERIALS AND METHODS

After institutional review board approval, we prospectively followed men who underwent microsurgical varicocelectomy from December 2018 to September 2019. Indications for varicocelectomy were according to the AUA guidelines.^14^ Varicocele was diagnosed by physical exam, and in those cases when clinical diagnosis was difficult (previous scrotal surgery or tight scrotum with difficult body habitus), scrotal ultrasound was performed. All subclinical varicoceles were excluded from this study. All surgeries were performed by a single high-volume surgeon, using microsurgical subinguinal technique. For all men, we evaluated serum 17-OHP, total T, FSH, and semen parameters before and after varicocele repair. All men had at least two semen analyses before undergoing varicocele repair according to the WHO guidelines. Men with azoospermia and severe oligozoospermia (concentration < 5 million/cc) in baseline had genetic evaluation prior to treatment. We excluded patients with chromosome abnormalities and microdeletions. Post-varicocelectomy measurements were obtained approximately 3–6 months after surgery. Three semen parameters were considered during the analysis: Concentration (million/cc), Motility (%), and Total Motile Sperm Count (TMSC). All semen analyses were performed by the same technician.

In order to develop a “standard” level for serum 17-OHP, we performed a cross-sectional analysis of a representative sample of 181 fertile controls (men who were evaluated for vasectomy reversal, erectile dysfunction, Peyronie’s disease, orchialgia, and vasectomy evaluation) aged 39 + 11.2 years. The 25th, 50th, and 75th percentile for serum 17-OHP was 55, 72, and 102 ng/dL. We used 55 ng/dL as the threshold to determine the lower limit of normal. The lower limit of normal established by commercial laboratories ranges from 27–199 ng/dL (LabCorp-www.labcorp.com) and from 33–195 ng/dL (QUEST—www.questdiagnotics.com). We preferred to use the cut point of 55 ng/dL, because we wanted to use a representative sample of fertile controls and was similar to the cutoffs used by the large commercial laboratories. Therefore, we evaluated men based on serum 17-OHP < 55 ng/dL (low 17-OHP) or 17-OHP > 55 ng/dL (normal 17-OHP). Also, patients were divided according to baseline total motile sperm count (TMSC) in two groups: TMSC < 9 and TMSC > 9. We excluded men without baseline and post-varicocelectomy measurements, those receiving exogenous T.

### 2.1 | Statistical analysis

Statistical analysis was performed with SPSS version 24.0 software. Categorical variables were presented as absolute values and frequencies, categorical variables were analyzed with a Chi-square test. For continuous variables, means, and standard deviations (± SD) or medians and interquartile ranges [25–75] were calculated according to the data distribution. Comparison of numerical variables between groups was performed using the U Mann-Whitney, Student’s *t* test and Wilcoxon rank test. Correlation analysis was performed with the Spearman test. A P-value < .05 was considered statistically significant.

## 3| RESULTS

Between December 2018 and September 2020, a total of 30 men fit the inclusion criteria and underwent varicocelectomy. Clinical and demographic characteristics of the analyzed men are evident in Table 1. In the baseline analysis, 6 (20%) of men had T < 300 ng/ dL while 24 (80%) men had T > 300 ng/dL. Also, 9 (30%) men had 17-OHP < 55 ng/dL and 21 (70%) men presented with 17-OHP > 55 ng/dL. Of the nine men with low 17-OHP, only two (22.2%) presented with orchialgia. This is a very similar proportion to men with normal 17-OHP, where five (23.8%) presented with orchialgia. When divided according to TMSC, 19 men had TMSC < 9 million, including 6 azoospermic and 1 cryptozoospermic, and 11 men with TMSC > 9 million (Table 2). Of the 19 patients with baseline TMSC < 9, 11 men had increase in TMSC and 7 men improved SA to TMSC > 9 in the follow-up. Conversely, only one man with baseline TMSC > 9 had a considerable decrease in TMSC to < 9. Men with TMSC < 9 after surgery had higher levels of FSH in baseline and follow-up, higher prevalence of bilateral varicocele, smaller testicles, and lower TMSC count in the baseline (Table 2).

When analyzing serum hormones changes after surgery, there was no significant difference of 17-OHP, FSH, LH, and T levels when compared to baseline (*P* > .05). In contrast, most SA parameters had significant improvement after surgery, including volume (2.1 [1.52.8] to 2.4 [1.7–3.6] cc, *P* = .018), concentration (6.8 [0.8–22.5] to 12.5 [1–31], *P* = .047) and TMSC (4.4 [0.3–15.1] to 10.5 [0–41.8], *P* = .012) (Table 3). When evaluating results after diving men according to baseline serum 17-OHP < 55 or > 55 ng/dL, there was no difference in follow-up semen parameters (*P* > .05) (Table 4). When considering T levels, those with T < 300 showed improvement of T levels of 251 [190–276.5] ng/dL to 362 [194.3– 483] ng/dL (*P* = .019), while those with T > 300 had change on T of 518.5 [458–627.5] ng/dL to 505 [406.3–624.5] ng/dL (*P* = .046).

After performing a correlational analysis, we found a positive correlation between baseline T with baseline 17-OHP (r = .691, *P* < .001), follow-up T and follow-up 17-OHP (r = .400, *P* = .029) and follow-up 17-OHP and postoperative semen volume (r = .429, *P* = .018). But, we did not find significant correlation between preoperative 17-OHP and SA concentration, TMSC, varicocele grade, and laterality (*P* > .05) (Table 5).

We also evaluated the effected of higher grades of varicocele in hormonal levels and semen parameters by excluding grade I varicocele from the analysis. Only TMSC variation was significant in this particular group (4.85 [0.55–22.25] to 11 [1.05–53] million, *P* = .028) (Table 6).

## 4| DISCUSSION

Varicocele has been proposed to be the cause of infertility in a considerable amount of men seeking to conceive. Varicocelectomy is a cost-effective treatment for infertility, with microsurgical subinguinal or inguinal as the best techniques.^15^ Since many studies showed correlation between varicocele repair and serum T levels improvement,^7–10,16^ we considered the hypothesis of varicocele impairment in Leydig cell function and detriment in ITT levels to proceed with this study. Therefore, we analyzed the correlation between varicocele repair and semen parameters, ITT using 17-OHP and serum T postoperative changes.

In our study on 30 patients, sperm concentration, motility, and TMSC improved in 60%, 46.6%, and 60% of patients. Using the Samplaski et al^17^ concept of semen upgrade, 7 (36.8%) men with TMSC < 9 ameliorated TMSC to > 9 and reached natural conception parameters, proving that varicocele repair is an important tool in the andrologist arsenal. Although many studies showed that varicocele repair could improve T levels, we could not reproduce these finding in our study. Also, there was no significant correlation between serum 17-OHP and semen analysis improvement after the surgery. Therefore, we could not verify our initial hypothesis.

Previous studies showed that men with normal T are less likely to improve total T postoperatively.^18^ These findings come in agreement with our population, since men with low 17-OHP levels showed improvement in both serum 17-OHP (increase of 28 [16–30] ng/dL) and T levels (increase of 58 [43–178] ng/dL), while men with normal 17- OHP had a decrease in those hormones.

Our study not only has strengths, but it also has its limitations. The strengths include the novelty of the study, associating varicocelectomy with ITT and semen parameters. All procedures were performed using microsurgical technique by a single high-volume surgeon and all semen analyses were performed by a single specialist, reducing bias in surgical technique as well as sperm analyses. We also tried to control variability in this study by performing all serum measurements between 6:00 and 10:00 am, in a single laboratory. Although we could not define a precise association between ITT and SA parameters improvement after varicocelectomy, our findings of concentration and TMSC improvement after surgery comes in agreement with other studies,^19,20,21^ giving validation to our study. Furthermore, we performed follow-up assessment with adequate time. However, this is a single institution study with a modest sample size and our findings should be validated in larger population cohort. Although the primary outcome was to evaluate changes in intratesticular T and semen parameters after surgery, we did not evaluate effects in pregnancy and live birth rates. Also, we based the correlation of 17-OHP with ITT in Amory et al^12^ findings, previously tested by Lima et al,^13^ since repeatedly performing testicular aspiration was not feasible.

Medical therapy for infertile men with low T is well described in the literature. However, noninvasive assessment of ITT and its use in practice remains poorly described. Serum 17-OHP could be used to titrate medical therapy of men,^13^ hypogonadotropic hypogonadism,^22^ and men with oligozoospermia^23^ and low T. In contrast, baseline 17-OHP could not predict semen analysis improvement after varicocele repair, and its use for this purpose should be investigated in a larger scale for selected patients, such as those with clinical varicocele and baseline low T levels but cannot be used at this time as a biomarker to predict sperm parameter improvement after varicocele repair. Future studies to evaluate biomarkers to predict sperm parameter improvement after varicocele repair should utilize pathways other than androgen synthesis.

In conclusion, microsurgical varicocele repair resulted in improvements in all evaluated semen parameters, but not in ITT/17-OHP or serum T levels. The mechanism by which varicocele repair improves semen parameters appears to be driven by factors other than changes in androgen synthesis within the testicular microenvironment.

## Figures and Tables

**TABLE 1:** Clinical and demographic characteristics of the analyzed patients

Characteristics	Patients n = 30
Age at surgery (years)	34.1 ± 8.1
Mean testicular volumen (cc)	13.6 ± 3.2
Highest varicocele grade	
I	8 (26.7%)
II	15 (50%)
III	7 (23.3%)
Varicocele laterality	
Unilateral (Left)	13 (43.3%)
Bilateral	17 (56.7%)
Indication for surgery	
Infertility	23 (76.7%)
Orchialgia	7 (23.3%)
Time from surgery to blood work (months)	3.8 [3.6–7.7]
Time from surgery to SA (months)	3.7 [3.5–6.5]

*Note:* Mean ± standard deviation, Median [interquartile range 25–75].

**TABLE 2:** Comparison of the clinical variables between the patients that had a TMCS > and < than 9 on follow-up after varicocelectomy

	TMCS after varicocelectomy		
	≥9 n = 17	<9 n = 13	*P*-value
Age at surgery (years)	32.5 ± 7.4	36.2 ± 8.8	.230
Mean testicular volume (cc)	15 ± 2.9	11.9 ± 2.7	**.006**
Highest varicocele grade			
I	3 (17.6%)	5 (38.5%)	
II	9 (52.9%)	6 (46.2%)	
III	5 (29.4%)	2 (15.4%)	.389
Varicocele laterality			
Unilateral (Left)	10 (58.8%)	3 (23.1%)	
Bilateral	7 (41.2%)	10 (76.9%)	**.050**
Baseline TMSC	10.5 [4.9–23.5]	0 [0–0.90]	**<.001**
Baseline 17-OHP	95 [46.5–146.5]	71 [42–131]	.691
Baseline T	489 [347.5–653.5]	470 [303.5–613.5]	.851
Baseline FSH	3.9 [3.5–6.4]	9.3 [7–14]	**<.001**
Baseline LH	3.9 [3.4–7.2]	5 [3.2–8.6]	.506
Follow-up 17-OHP	72 [46–117.5]	62 [53.5–116]	.983
Follow-up T	441 [329–568]	506 [409.5–635]	.391
Follow-up FSH	4.3 [2.9–6.8]	8.4 [6.3–15.3]	**.004**
Follow-up LH	3.9 [2.6–7.2]	4.5 [3.1–7.3]	.598

*Note:* Mean ± standard deviation, median [interquartile range 25–75].

Abbreviations: 17-OHP: 17-hydroxyprogesterone (ng/dL); FSH: Follicle-stimulating hormone (mIU/mL); LH: Luteinizing hormone (mIU/mL); SA: Semen analysis; T: Testosterone (ng/dL); TMSC: Total motile sperm count (million).

**TABLE 3:** Comparison between hormonal levels and semen analysis parameters at baseline and after the procedure

	Baseline n = 30	Follow-up n = 30	*P*-value
Hormones			
17-OHP ng/dL	85 [47.3–141.8]	67 [51.8–116.3]	.185
T	489 [328.5–610.3]	463 [348–588.5]	.267
FSH	6.5 [3.8–10.2]	6.3 [3.6–11.2]	.657
LH	4.7 [3.4–7.4]	4.5 [2.7–7.3]	.124
Semen analysis			
Volume	2.1 [1.5–2.8]	2.4 [1.7–3.6]	**.018**
Sperm concentration	6.8 [0.8–22.5]	12.5 [1–31]	**.047**
Total motility	22 [7.5–44.8]	33 [0–50]	.201
TMSC	4.4 [0.3–15.1]	10.5 [0–41.8]	**.012**

*Note:* Median [interquartile range 25–75].

Abbreviations: 17-OHP: 17-hydroxyprogesterone (ng/dL); FSH: Follicle-stimulating hormone (mIU/mL); LH: Luteinizing hormone (mIU/mL); SA: Semen analysis; T: Testosterone (ng/dL); TMSC: Total motile sperm count (million).

**TABLE 4:** Comparison between hormonal levels and semen analysis parameters at baseline and after the procedure in accordance to the 17 OHP value

	Baseline 17-OHP		
	≥55 ng/dL (n = 21)	<55 ng/dL (n = 9)	*P*-value
Follow-up hormones			
T	473 [401–600]	453 [296–599]	.603
FSH	5 [3.6–9.7]	7 [4.6–13.1]	.390
LH	4.5 [2.7–7.3]	4.4 [2.6–8.1]	.807
Follow-up SA			
Volume	2.6 [1.9–3.7]	1.7 [1.6–3.5]	.123
Sperm concentration	13 [3.2–32]	12 [0.1–33]	.838
Total motility	33 [6.5–50]	23 [0–58]	.766
TMSC	10 [0.7–44.5]	11 [0–37.5]	.698
	**Follow-up 17-OHP**		
	**≥55 ng/dL (n = 20)**	**<55 ng/dL (n = 10)**	
Follow-up hormones			
T	516 [420.3–639.5]	363 [258.5–521.3]	**.043**
FSH	5.1 [3.2–12.8]	7 [4.8–11.2]	.311
LH	4.7 [3.1–7.6]	3.5 [2.6–5.5]	.370
Follow-up SA			
Volume	2.7 [2–3.7]	1.6 [1.6–3.7]	.064
Sperm concentration	12.5 [0.3–31.5]	13 [5.3–34]	.627
Total motility	33 [0–53]	30.5 [15.8–46.5]	.912
TMSC	10 [0–55.8]	11 [1.8–41.8]	.982

*Note:* Median [interquartile range 25–75].

Abbreviations: 17-OHP: 17-hydroxyprogesterone (ng/dL); FSH: Follicle-stimulating hormone (mIU/mL); LH: Luteinizing hormone (mIU/mL); SA: Semen analysis; T: Testosterone (ng/dL); TMSC: Total motile sperm count (million).

**TABLE 5:** Correlation between 17-OHP before and after varicocelectomy and clinical, hormonal, and semen parameters at baseline and after procedure (Spearman rho)

		Baseline 17-OHP		Follow-up 17-OHP	
Variable	Time	*r*	*P*-value	*r*	*P*-value
Age at surgery		−.269	.151	−.238	.206
Testicular volume		−.339	.072	−.331	.080
Varicocele grade		−.123	.519	.033	.863
Varicocele laterality		.074	.698	−.121	.526
T	Baseline	.691	**<.001**	.400	**.029**
	Follow-up	.311	.094	.501	**.005**
FSH	Baseline	−.115	.553	−.124	.521
	Follow-up	−.286	.125	−.200	.289
Volume	Baseline	−.148	.442	.055	.777
	Follow-up	.256	.172	.429	**.018**
Sperm concentration	Baseline	.313	.092	−.099	.602
	Follow-up	.032	.866	−.082	.668
Total motility	Baseline	.128	.502	−0.134	.482
	Follow-up	.015	.937	−0.086	.652
TMSC	Baseline	.216	.251	−0.110	.562
	Follow-up	.037	.845	0.039	.840

Abbreviations: 17-OHP: 17-hydroxyprogesterone; FSH: Follicle-stimulating hormone; TMSC: Total motile sperm count.

**TABLE 6:** Comparison between hormonal levels and semen parameters at baseline and after procedure in men with grade 2 and 3 varicoceles

	Baseline n = 22	Follow-up n = 22	*P*-value
Hormones			
17-OHP	89.5 [29.5–141.8]	48 [56.5–100]	.168
T	502.5 [328.5–598.5]	478.5 [348–574]	.363
FSH	5.9 [3.7–9.3]	6 [3.3–12]	.411
LH	4.4 [2.8–7.2]	3.9 [2.6–6.3]	.258
Semen analysis			
Volume	2 [1.3–2.8]	2.2 [1.6–3.6]	.179
Sperm concentration	8.25 [1.75–24.75]	13.5 [5.55–35.5]	.061
Total motility	29.5 [14.5–51]	42 [12.75–51]	.327
TMSC	4.85 [0.55–22.25]	11 [1.05–53]	**.028**

*Note:* Median [interquartile range 25–75].

Abbreviations: 17-OHP: 17-hydroxyprogesterone (ng/dL); FSH: Follicle-stimulating hormone (mIU/mL); LH: Luteinizing hormone (mIU/mL); SA: Semen analysis; T: Testosterone (ng/dL); TMSC: Total motile sperm count (million).
